# Supplementary material for: MetaRibo-Seq measures translation in microbiomes
Source: Nat Commun. 2020 Jun 29;11:3268. doi: 10.1038/s41467-020-17081-z (PMC7324362; doi:10.1038/s41467-020-17081-z)
Supplement: Supplementary file 10 — Supplementary Data 7 [file 41467_2020_17081_MOESM10_ESM.zip › File2/Confidence_High_Taxonomy/409919_out.krona.html]

Javascript must be enabled to view this page.

members
magnitude
magnitudeUnassigned
count
unassigned
taxon
rank

409919\_out

95

superkingdom
95
2

phylum
976
95

95
200643
class

171549
95
order

family
1853231
2

2
283168
genus

species
2
28118

SRS053398\_contig\_number\_12812SRS098571\_contig\_number\_18779

815
93
family


SRS017821\_contig\_number\_24117SRS050801\_contig\_number\_1213SRS056537\_contig\_number\_40464
93
genus
3
816


SRS021153\_contig\_number\_1043SRS049446\_contig\_number\_6728SRS075773\_contig\_number\_27196SRS075821\_contig\_number\_3322
820
4
species

1
28116

SRS014683\_contig\_number\_7618
species

species
818

SRS018817\_contig\_number\_250
1

species
62
821

SRS013382\_contig\_number\_722SRS014287\_contig\_number\_2912SRS014534\_contig\_number\_353SRS014948\_contig\_number\_18266SRS015369\_contig\_number\_298SRS015486\_contig\_number\_403SRS016056\_contig\_number\_537SRS016132\_contig\_number\_865SRS016989\_contig\_number\_5378SRS017191\_contig\_number\_2650SRS017307\_contig\_number\_14567SRS019068\_contig\_number\_2917SRS019910\_contig\_number\_20414SRS020622\_contig\_number\_6159SRS020869\_contig\_number\_14575SRS021948\_contig\_number\_4093SRS043411\_contig\_number\_11220SRS043701\_contig\_number\_2428SRS046810\_contig\_number\_7SRS049773\_contig\_number\_30935SRS050752\_contig\_number\_29008SRS051031\_contig\_number\_23722SRS053087\_contig\_number\_2487SRS053335\_contig\_number\_3465SRS053356\_contig\_number\_2965SRS062427\_contig\_number\_1342SRS063928\_contig\_number\_439SRS064557\_contig\_number\_2754SRS075984\_contig\_number\_12283SRS077991\_contig\_number\_26SRS097889\_contig\_number\_26050SRS100907\_contig\_number\_8650SRS1041039\_contig\_number\_29002SRS1041091\_contig\_number\_16628SRS1041133\_contig\_number\_20120SRS1041138\_contig\_number\_3576SRS1041140\_contig\_number\_22260SRS1041144\_contig\_number\_2898SRS1054716\_contig\_number\_14970SRS1054928\_contig\_number\_1034SRS1055067\_contig\_number\_14269SRS1055076\_contig\_number\_contig-100\_1202.95615SRS142542\_contig\_number\_11861SRS142599\_contig\_number\_25664SRS143085\_contig\_number\_2177SRS143417\_contig\_number\_6694SRS143598\_contig\_number\_13356SRS143780\_contig\_number\_1775SRS143991\_contig\_number\_17738SRS146812\_contig\_number\_59562SRS147039\_contig\_number\_1431SRS147377\_contig\_number\_3792SRS147766\_contig\_number\_13759SRS148817\_contig\_number\_580SRS148970\_contig\_number\_1155SRS149325\_contig\_number\_12187SRS893292\_contig\_number\_6488SRS893342\_contig\_number\_8229SRS893358\_contig\_number\_9730SRS893369\_contig\_number\_7350SRS893378\_contig\_number\_10407SRS971275\_contig\_number\_2288

357276

SRS013940\_contig\_number\_4243SRS018049\_contig\_number\_189SRS024663\_contig\_number\_2058SRS045195\_contig\_number\_1355SRS052078\_contig\_number\_576SRS148874\_contig\_number\_5699
6
species

7
2292935

SRS014613\_contig\_number\_1435SRS048060\_contig\_number\_10003SRS064276\_contig\_number\_27285SRS1041145\_contig\_number\_13602SRS1054691\_contig\_number\_13471SRS1055043\_contig\_number\_11980SRS1055099\_contig\_number\_2067
species

species
1

SRS101268\_contig\_number\_28
817

species
246787

SRS098571\_contig\_number\_13815
1

species

SRS023526\_contig\_number\_26530SRS078242\_contig\_number\_455
46506
2

457391

SRS015264\_contig\_number\_10630SRS043001\_contig\_number\_1759SRS144753\_contig\_number\_12247
3
species

species
1
204516

SRS052027\_contig\_number\_10059

species
329854

SRS098073\_contig\_number\_11926
1
